# Use of prepectoral polyurethane coated implants in the setting of radiotherapy: the OPBC-09 PRExRT retrospective cohort study

**DOI:** 10.1016/j.breast.2026.104951

**Published:** 2026-10-30

**Authors:** Florian Fitzal, Rama Kiblawi, Kerstin Wimmer, Florian S. Halbeisen, Gianluca Franceschini, Lorenzo Scardina, Fabio Marazzi, Simona Gipe, Cynthia Kohl, Franziska Trapp, Maja Molska, Oliwia Bednarek, Martin Heidinger, Edvin Ostapenko, Amit Agrawal, Konstantina Balafa, Maggie Banys-Paluchowski, Justin M. Broyles, Oreste D. Gentilini, Maite Goldschmidt, Eduardo González, Martin Haug, Elisabeth A. Kappos, Michael Knauer, Giacomo Montagna, Ira Oikonomou, Martín Rivas Ibarra, Simone Ross-Keck, Dirk J. Schaefer, Andy Schneider, Nicole Speck, Dario Virzi, Mateusz Wichtowski, Artefaa Al-Shamari, Mowid Hamad, André Pfob, Jörg Heil, Lisa Stenman Skarsgård, Christian Korvald, Daniele Passeri, Mariacristina Toffanin, Massimo Ferrucci, Walter P. Weber, PRExRT Study group, Khaled Abdelwahab, Guldeniz Karadeniz Cakmak, Francesco Caruso, Sabatino D'Archi, Rosa Di Micco, Annasilvia Di Pumpo, Salahddin A. Gehani, Camila Gutierrez Neira, Laura Henderson, Tari A. King, Jaime Letzkus Berrios, Maciej Łukaszek, Zoltan Matrai, Petra Palkó, Eleonora Petrazzuolo, Valentina Pezzetti, Pier Carlo Rassu, Anne Marie Raymakers, Nicola Rocco, Nicole Rotmensz, Tina Schnitzbauer, Nikolas Tauber, José H. Volders, Denise Vorburger

**Affiliations:** ak Mansoura Oncology Center, Surgical Oncology Department, Faculty of Medicine, Mansoura University, Mansoura, Egypt; al Department of General Surgery, School of Medicine, Zonguldak Bulent Ecevit University, Zonguldak, Turkey; am Breast Surgery Unit, Humanitas Istituto Clinico Catanese, Misterbianco, Catania, Italy; an Breast Unit, Department of Women and Children Health Sciences, Fondazione Policlinico Universitario Agostino Gemelli IRCCS, Italy; ao Breast Surgery, IRCCS Ospedale San Raffaele, Milan, Italy; ap Surgical Department, Breast Oncoplastic Unit, Hamad Medical Corporation, Doha, Qatar; aq Department of Breast Surgery, Arturo López Pérez Foundation, Santiago, Chile; ar Department of Breast Surgery, Cambridge University Hospitals, Cambridge, UK; as Division of Breast Surgery, Brigham and Women's Hospital, Dana Farber/Brigham Cancer Center, Boston, MA, USA; at Breast Surgical Unit, San Borja Arriarán Clinical Hospital-Clínica MEDS, Santiago, Chile; au Department of Surgical Oncology, Institute of Oncology, Poznan University of Medical Sciences, Poznan, Poland; av Department of Obstetrics and Gynecology, Heidelberg University Hospital, Heidelberg, Germany; aw Breast Center Heidelberg, Hospital St. Elisabeth, Heidelberg, Germany; ax General Surgery Department, San Giacomo Hospital, Novi Ligure, Alessandria, Italy; ay Division of Plastic and Reconstructive Surgery, Brigham and Women's Hospital, Boston, MA, USA; az Department of Plastic and Reconstructive Surgery, University Medical Center Groningen, the Netherlands; ba Department of Advanced Biomedical Sciences, University of Naples Federico II, Naples, Italy; bb Interdisciplinary Breast Center, Kliniken Essen-Mitte, Essen, Germany; bc Department of Gynecology and Obstetrics, University Hospital Schleswig-Holstein, Campus Lübeck, Lübeck, Germany; bd Department of Surgical Oncology, Diakonessenhuis, Utrecht, the Netherlands; be Breast Cancer Center, Department of Gynecology, University Hospital Zurich, Zurich, Switzerland; a Department of General Surgery and Breast Health Center, Hanusch Hospital, Vienna, Austria; b ABCSG, Austrian Breast and Colorectal Cancer Study Group, Vienna, Austria; c Breast Clinic, University Hospital Basel, Basel, Switzerland; d University of Basel, Basel, Switzerland; e Department of Gynecology & Obstetrics, University Hospital Basel, Basel, Switzerland; f Department of General Surgery, Division of Visceral Surgery and Comprehensive Cancer Center, Medical University of Vienna, Vienna, Austria; g Surgical Outcome Research Center, University Hospital Basel and University of Basel, Basel, Switzerland; h Breast Unit, Department of Women and Children Health Sciences, Fondazione Policlinico Universitario Agostino Gemelli IRCCS, Rome, Italy; i Department of Radiation Oncology, Fondazione Policlinico Universitario Agostino Gemelli IRCCS, Rome, Italy; j Interdisciplinary Breast Center, Kliniken Essen-Mitte, Essen, Germany; k University Department of Obstetrics and Gynecology, Clinic St. Hedwig of the Order of St. John, University of Regensburg, Germany; l Clinical Department of General and Oncological Surgery, University Hospital in Zielona Góra, Zielona Góra, Poland; m Department of Oncological Surgery, Collegium Medicum, University of Zielona Góra, Poland; n Breast Center, OST KLINIKA, Vilnius, Lithuania; o Department of Breast Surgery, Cambridge University Hospitals, Cambridge, UK; p Department of Breast Surgery, University of Cambridge, Cambridge, UK; q Breast Surgery Unit, Humanitas Istituto Clinico Catanese, Misterbianco, Catania, Italy; r Department of Gynecology and Obstetrics, University Hospital Schleswig-Holstein, Campus Lübeck, Lübeck, Germany; s University of Luebeck, Luebeck, Germany; t Division of Breast Surgery, Brigham and Women's Hospital, Dana Farber/Brigham Cancer Center, Boston, MA, USA; u Breast Surgery Unit, IRCCS Ospedale San Raffaele, Milan, Italy; v Vita-Salute San Raffaele University, Milan, Italy; w Mastology Unit and Department of Mastology, Sanatorio Modelo Quilmes, University of Buenos Aires, Argentina; x Department of Plastic, Reconstructive, Aesthetic and Hand Surgery, University Hospital Basel, Basel, Switzerland; y Tumor and Breast Center Eastern Switzerland, St. Gallen, Switzerland; z Breast Service, Department of Surgery, Memorial Sloan Kettering Cancer Center, New York, NY, USA; aa Department of Surgery, Division of Breast Surgery, Södersjukhuset, Stockholm, 118 83, Sweden; ab Department of Clinical Science and Education Södersjukhuset, Karolinska Institutet, Stockholm, Sweden; ac Department of Breast Surgery, Arturo López Pérez Foundation, Santiago, Chile; ad Plastic Surgery Unit, Humanitas Istituto Clinico Catanese, Misterbianco, Catania, Italy; ae Department of Surgical Oncology, Institute of Oncology, Poznan University of Medical Sciences, Poznan, Poland; af Surgical Department, Breast Oncoplastic Unit, Hamad Medical Corporation, Doha, Qatar; ag Department of Obstetrics and Gynecology, Heidelberg University Hospital, Heidelberg, Germany; ah Breast Center Heidelberg, Hospital St. Elisabeth, Heidelberg, Germany; ai Department of Plastic and Reconstructive Surgery, Oslo University Hospital, Oslo, Norway; aj Breast Surgery Unit, Veneto Institute of Oncology IOV, Istituto di Ricovero e Cura a Carattere Scientifico (IRCCS), Padova, Italy

**Keywords:** Polyurethane coated implants, Breast cancer, Implant-based breast reconstruction, Radiotherapy, Breast surgery, Retrospective cohort study, OPBC

## Abstract

**Background:**

Radiotherapy (RT) increases the risk of capsular contracture after mastectomy with implant-based breast reconstruction (IBBR). The present study assessed the association between the use of polyurethane coated versus non-polyurethane coated implants and the likelihood of surgical revision due to capsular contracture after RT.

**Methods:**

The international multicenter retrospective PRExRT cohort study included patients with breast cancer treated at 26 sites in 15 countries between January 2016 and December 2024. Eligible patients underwent prepectoral IBBR with either polyurethane coated or non-polyurethane coated implants and received RT.

Primary endpoint was surgical revision due to capsular contracture and was analyzed using Cox regression models.

**Results:**

Among 1455 patients, 475 (32.6%) received polyurethane coated implants and 980 (67.3%) non-polyurethane coated implants.

The use of polyurethane coated implants was associated with less frequent use of synthetic or biologic mesh (1.6% vs. 42.8%, p < 0.001), and less frequent two-stage IBBR (8.4% vs. 21.5%, p < 0.001).

Compared to IBBR with non-polyurethane coated implants, use of polyurethane coated implants was independently associated with a lower rate of surgical revision due to capsular contracture (9.3% vs. 25.7%; HR 0.2, 95%CI 0.1-0.5, p < 0.001).

The risk of major infection (OR 0.3, 95%CI 0.2-0.6, p < 0.001), implant loss (OR 0.2, 95%CI 0.09-0.4, p < 0.001), reoperation (OR 0.3, 95%CI 0.2-0.6, p = 0.001) and implant rotation (OR 0.05, 95%CI 0.004-0.7, p = 0.02) was also lower in the polyurethane group.

**Conclusions:**

In the setting of RT, the use of prepectoral polyurethane coated implants was associated with a lower likelihood of surgical revision due to capsular contracture compared to non-polyurethane coated implants.

**Trial registration:**

ClinicalTrials.gov identifier: NCT06747065.

## Highlights

• Radiotherapy increases capsular contracture (CC) after implant-based reconstruction.
• Evidence on polyurethane-coated implants (PUc) after radiotherapy remains scarce.
• In this cohort, PUc implants were associated with fewer surgical revisions for CC.
• Other surgical complications were also significantly less frequent with PUc implants.
• These findings may inform planning in the setting of postmastectomy radiotherapy.

## Graphical abstract

**Figure:**
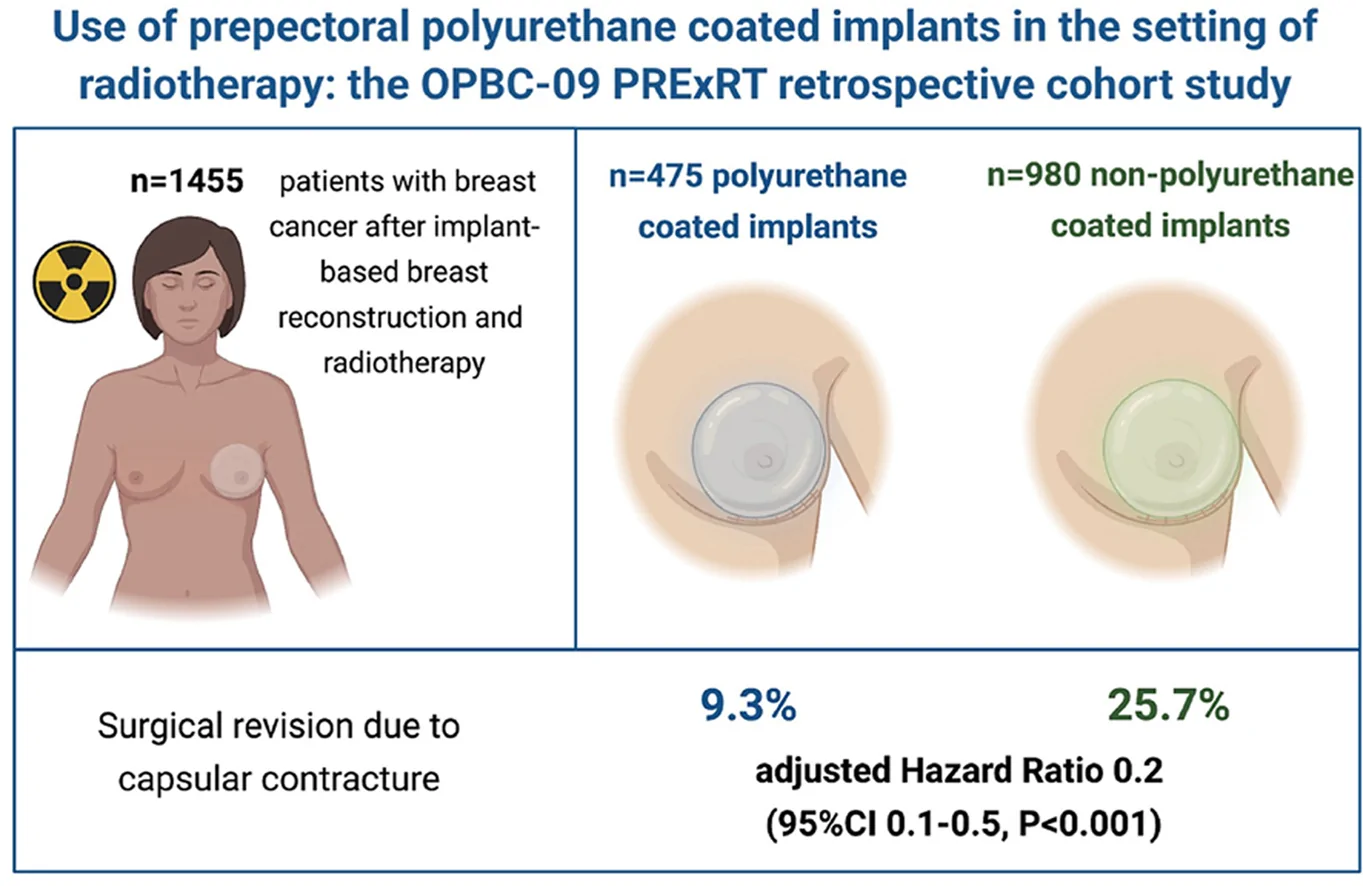

## Abbreviations

ADM: acellular dermal matrix
aHR: adjusted hazard ratio
aOR: adjusted odds ratio
BC: breast cancer
BMI: body mass index
CC: capsular contracture
IBBR: implant-based breast reconstruction
IQR: interquartile range
HER2: Human Epidermal Growth Factor Receptor 2
HR: hazard ratio
Non-PUc: non-polyurethane coated
NSM: nipple-sparing mastectomy
OR: odds ratio
p: p-value
PMRT: post-mastectomy radiation therapy
PUc: polyurethane coated
QoL: quality of life
RT: radiotherapy
SSM: skin-sparing mastectomy
STROCSS: Strengthening the reporting of cohort, cross-sectional and case-control studies in surgery
95% CI: 95% confidence interval

## 1 Introduction

Breast cancer (BC) has recently overtaken lung cancer as the most frequently diagnosed malignancy worldwide [1] and remains the leading cause of cancer-related death among women globally [2].

Despite major advances in early detection and treatment, mastectomy continues to be the surgical treatment of choice for an increasing proportion of patients.

This increase is largely attributed to more widespread use of breast magnetic resonance imaging (MRI) [3], enhanced access to genetic testing, and a growing patient preference for mastectomy as a risk-reducing or definitive surgical approach. Currently, approximately 30% to 40% of women diagnosed with BC undergo mastectomy [4], [5].

Breast reconstruction following mastectomy significantly improves quality of life (QoL), primarily by providing superior aesthetic outcomes [6], [7]. While autologous breast reconstruction is considered the optimal approach for many patients [8], [9], implant-based breast reconstruction (IBBR) remains the most frequently performed reconstructive approach [8]. At the same time, a growing proportion of patients require postmastectomy radiotherapy (PMRT). In line with expanding guideline indications (e.g. NCCN) [10], PMRT is currently administered to up to one-third of patients undergoing mastectomy [11], [12]. This poses a particular challenge for IBBR, as PMRT following IBBR negatively impacts QoL and is associated with increased rates of capsular contracture and implant failure [13], [14].

Over the last years, there has been a notable shift in IBBR, from subpectoral to prepectoral approach. By preserving the pectoralis major muscle, prepectoral IBBR avoids breast animation deformity and may provide a more natural breast contour and appearance [15], [16], [17]. It has also been associated with lower rates of capsular contracture [18]. Most recently, the randomized phase III PREPEC trial reported significantly better patient-reported physical well-being at 24 months after prepectoral compared with subpectoral IBBR [19]. This shift towards prepectoral IBBR has been supported by advances in implant surface technology, such as polyurethane coated implants [20]. Polyurethane coated implants integrate with the surrounding tissue within days after surgery, which limits implant movement without requiring mesh or acellular dermal matrix (ADM) support [21]. In addition, they have shown the lowest rates of rippling and capsular contracture up to 10 years after surgery [22], [23].

Although prior retrospective studies suggest a lower risk of capsular contracture with polyurethane coated implants [24], [25], [26], we aimed to evaluate whether prepectoral reconstruction with polyurethane coated implants results in lower rates of capsular contracture and implant loss compared to non-polyurethane coated implants in the setting of RT in a large multicenter study.

## 2 Methods

### 2.1 Design and study population

The Oncoplastic Breast Consortium (OPBC)-09/PRExRT retrospective cohort study included patients from 26 sites across 15 countries and 5 continents who were treated from 1 January 2016 to 31 December 2022 (Supplementary Table 1), with follow-up until 31 December 2024. The sites comprised university and other public teaching hospitals, as well as private sites of varying sizes and district general hospitals. Data were collected from institutional databases, registries or patient charts. Informed consent was sought in accordance with ethical approval that was study-/database-specific or based on general consent, in accordance with site-specific and national standards. A data use agreement was executed between each participating site and the leading site, which served as coordinating center. The principal investigator at each site was responsible for data collection and transfer, whereas data cleaning and analysis were conducted at the leading site. The data analysis was performed from October to December 2025. This cohort study has been reported in line with the Strengthening the reporting of cohort, cross-sectional and case-control studies in surgery (STROCSS) guidelines [27].

Patients with early-stage BC (pT0-T4, pN0-N3) or locally recurrent BC - following breast conserving surgery and radiotherapy (RT) - who underwent skin-sparing mastectomy (SSM), nipple-sparing mastectomy (NSM), or skin-reducing NSM were eligible. All patients underwent prepectoral IBBR using either polyurethane coated or non-polyurethane coated implants, and received RT. The majority of patients received PMRT to the definitive implant, the remaining patients were irradiated before definitive IBBR, either in case of recurrent breast cancer as adjuvant RT after prior breast-conserving surgery or in case of two-stage IBBR as PMRT to the expander. Additionally, utilization of synthetic meshes and acellular dermal matrices (ADM) was assessed. Exclusion criteria were stage IV disease, subpectoral IBBR, autologous reconstruction as initial type of reconstruction, omission of RT as well as missing follow-up (Fig. 1).

**Fig. 1:**
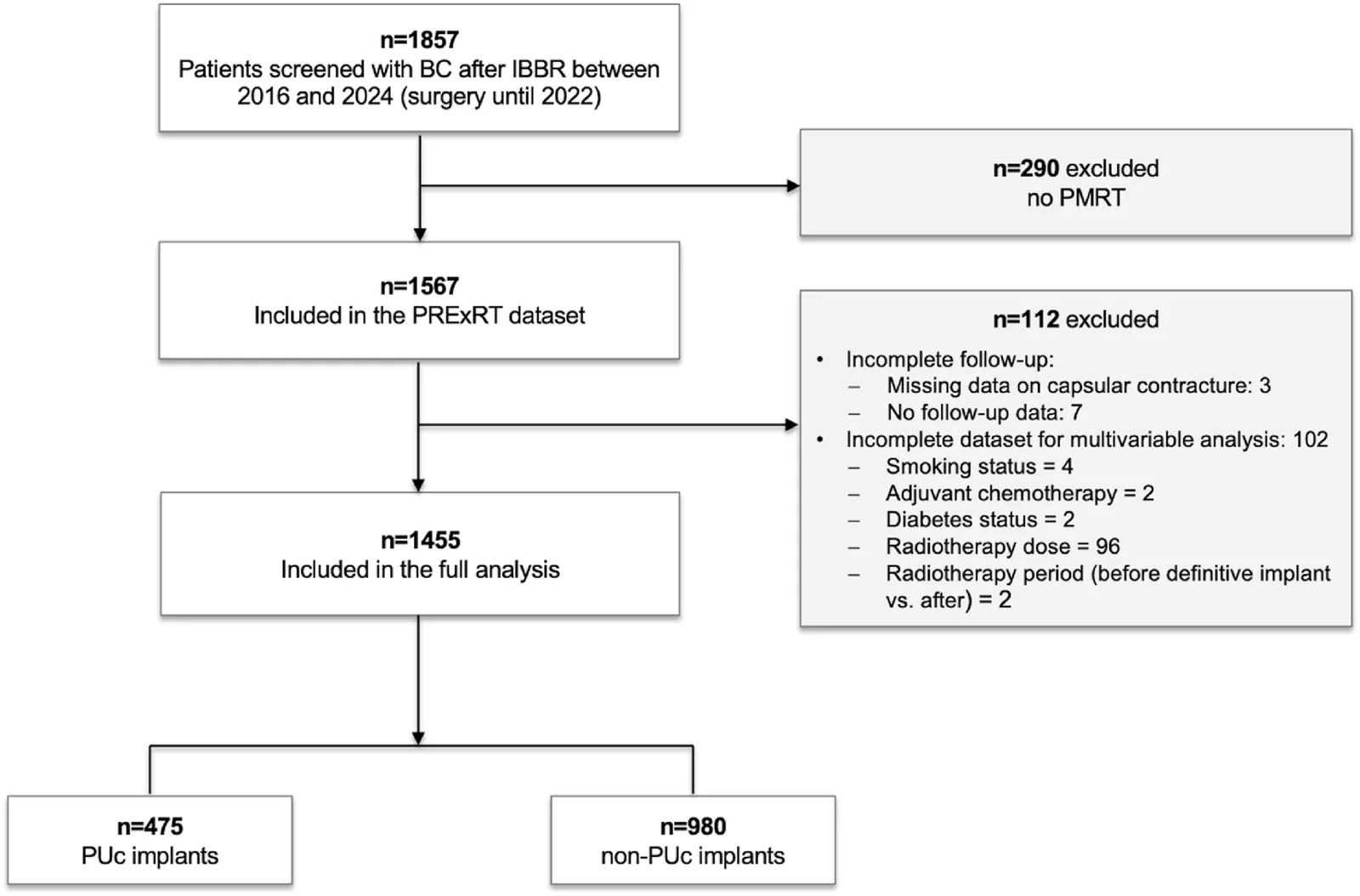
CONSORT diagram Abbreviations: BC, breast cancer; IBBR, implant-based breast reconstruction; PMRT, post-mastectomy radiotherapy; PUc, polyurethane coated Note: Reasons for exclusion are not mutually exclusive; therefore, totals may not equal the sum of individual reasons.

### 2.2 Type of breast implant

Eligible patients were divided into two groups: those receiving a polyurethane coated implant and those receiving a non-polyurethane coated implant (Supplementary Table 2a and 2b). Breast implant type was further classified according to texture, form and volume. These specifications were retrieved from surgical reports and from the implant manufacturer catalog.

### 2.3 Endpoints

The primary endpoint was the likelihood of surgical revision due to capsular contracture, stratified by implant type (polyurethane coated vs. non-polyurethane coated). Only re-operation with the explicit indication of capsular contracture was defined as an event. Data on subsequent surgical procedures were retrospectively collected and reported by the investigators at each participating center based on the information available in their clinical records. Procedures performed at other institutions were captured only if documented in these records.

Secondary endpoints included the odds of any capsular contracture and surgical complications. The following surgical complications were assessed: implant loss was defined as unplanned surgical removal of the expander or implant for any reason or duration with or without immediate replacement. Minor infection was defined by the need for oral antibiotic treatment, and major infection was defined by the need for surgery or intravenous antibiotic treatment. Clinically relevant hematoma and seroma were defined by the need for interventional or surgical evacuation. Wound healing disorders were registered when topical wound care or dressing changes were performed for more than 3 weeks. Further complications included skin necrosis, unplanned redo surgery, implant exposure, any rotation or displacement of the implant requiring repositioning under general anesthesia, and rippling.

### 2.4 Statistical analysis

No formal a priori sample size calculation was performed owing to the retrospective observational nature of the study. The sample size was determined by all consecutive eligible patients within the predefined study period. However, the achieved sample size permitted estimation of the crude absolute difference in surgical revision rates due to capsular contracture with reasonable precision, with a 95% confidence interval width of approximately 7.6 percentage points.

Descriptive statistics were used to summarize patient, tumor, and treatment characteristics. Categorical variables are presented as frequencies and percentages, while continuous variables are presented as medians with interquartile ranges (IQR). Comparisons of categorical variables between polyurethane coated and non-polyurethane coated-groups were performed using Fisher's exact test, while comparisons of continuous variables were conducted using the Wilcoxon rank-sum test.

Time-to-event outcomes were analyzed using mixed-effects Cox proportional hazards regression models including a random intercept for study center. Additional stratified analyses by one-vs. two-stage reconstruction were performed also with Cox proportional hazards regression models. Proportional hazards assumptions were assessed using scaled Schoenfeld residuals. Associations with binary outcomes for which no time-to-event data were available were examined using mixed-effects logistic regression including a random intercept for center. All multivariable models were adjusted for predefined clinical and treatment characteristics, including age at surgery, smoking status, diabetes mellitus status, radiotherapy period (before or after definitive IBBR, e.g. in case of two-stage IBBR), radiotherapy dose, and receipt of chemotherapy.

Contracture-free survival was estimated using the Kaplan-Meier method and stratified by implant type.

Additionally, multivariable Cox proportional hazards regression analysis was performed to evaluate factors associated with surgical revision due to CC.

Missing data were not imputed. Analyses were performed using complete-case data, with the available dataset for each respective outcome.

As a sensitivity analysis, propensity score weighting was performed to account for baseline differences between implant groups.

The results from the Cox regression model were described by means of hazard ratios (HRs), 95% confidence intervals (CIs) and p-values (p). Results were considered statistically significant with p < 0.05.

All statistical analyses were performed using R statistical software (Version 4.3.2, The R Foundation for Statistical Computing, Vienna, Austria).

Moreover, we declare that no artificial intelligence tool was employed in any phase of the study or development of the manuscript.

## 3 Results

Data from 1857 patients were collected, of whom 1567 were included in the study data set and 1455 in the full analysis set (Fig. 1). 475 (32.6%) patients had received a polyurethane coated implant and 980 (67.3%) a non-polyurethane coated implant.

The baseline characteristics stratified by type of implant are listed in Table 1. The median age at surgery was 47 (IQR 40-54) years. The majority of patients underwent unilateral surgery for invasive BC (92.7%) with pathologically T1 or T2 (67.5%) tumor stage and positive lymph nodes (61.8%). Patients who received polyurethane coated implants were more frequently smokers (18.1% vs. 10.0%, p < 0.001).

**Table 1:** Patient, tumor, and treatment characteristics.

	Overall N = 1455	PUc implants N = 475	Non-PUc implants N = 980	p-value
**Patient characteristics**				
**Follow-up time (years)**, median (IQR)	2.5 (1.3-3.5)	2.8 (1.5-3.6)	2.2 (1.2-3.5)	<0.001
**Age at surgery**, median (IQR)	47 (40-54)	47 (41-54)	47 (40-53)	0.08
**BMI (kg/m^2^)**, median (IQR)	24.4 (22.0-27.6)	24 (22-27)	24.6 (22.1-27.9)	0.01
**Smoking status**				<0.001
Never	1140 (78.3%)	332 (69.9%)	808 (82.4%)	
Ongoing	184 (12.6%)	86 (18.1%)	98 (10.0%)	
Ever	131 (9.0%)	57 (12.0%)	74 (7.5%)	
**Diabetes mellitus Type I or II**				0.08
Yes	68 (4.7%)	29 (6.1%)	39 (4.0%)	
No	1387 (95.3%)	446 (93.9%)	941 (96.0%)	
**Tumor characteristics**				
**Cancer classification**^a^				0.5
In situ (DCIS)	68 (4.7%)	19 (4.0%)	49 (5.0%)	
Invasive	1349 (92.7%)	439 (92.4%)	910 (92.9%)	
None/other	17 (1.1%)	1 (0.2%)	16 (1.6%)	
Missing	21 (1.4%)	16 (3.4%)	5 (0.5%)	
**Pathological tumor stage (pT/ypT)**^b^				<0.001
Tx	95 (6.5%)	29 (6.1%)	66 (6.7%)	
T1/T2	982 (67.5%)	365 (76.8%)	617 (63.0%)	
T3/T4	261 (17.9%)	48 (10.1%)	213 (21.7%)	
Missing	117 (8.0%)	33 (6.9%)	84 (8.6%)	
**Pathological nodal stage (pN/ypN)**^b^				0.7
Nx	25 (1.7%)	8 (1.7%)	17 (1.7%)	
N0	506 (34.8%)	173 (36.4%)	333 (34.0%)	
N1	643 (44.2%)	200 (42.1%)	443 (45.2%)	
N2/N3	256 (17.6%)	79 (16.6%)	177 (18.1%)	
Missing	25 (1.7%)	15 (3.2%)	10 (1.0%)	
**Ki-67, median (IQR)**	25 (12-42)	20 (10-40)	30 (15-45)	<0.001
**Surgical characteristics**				
**Mastectomy**				<0.001
Skin-sparing mastectomy	656 (45.1%)	89 (18.7%)	567 (57.9%)	
Nipple-sparing mastectomy	722 (49.6%)	343 (72.2%)	379 (38.7%)	
Skin-reducing nipple-sparing mastectomy	72 (4.9%)	43 (9.0%)	29 (3.0%)	
Missing	5 (0.3%)	0 (0.0%)	5 (0.5%)	
**Incision**				
Inverted T	163 (11.2%)	64 (13.5%)	99 (10.1%)	0.06
Submammary	541 (37.2%)	162 (34.1%)	379 (38.7%)	0.09
Radial	343 (23.6%)	190 (40.0%)	153 (15.6%)	<0.001
Periareolar	232 (15.9%)	51 (10.7%)	181 (18.5%)	<0.001
Radial-periareolar	104 (7.1%)	5 (1.0%)	99 (10.1%)	<0.001
Other	45 (3.1%)	7 (1.5%)	38 (3.9%)	0.01
Missing	32 (2.2%)	0 (0.0%)	32 (3.3%)	<0.001
**Synthetic mesh or acellular dermal matrix**				<0.001
Mesh or ADM	427 (29.4%)	8 (1.7%)	419 (42.8%)	
No Mesh or ADM	1025 (70.5%)	467 (98.3%)	558 (56.9%)	
Missing	3 (0.2%)	0 (0.0%)	3 (0.3%)	
**Implant texture among non-PUc**				
Smooth textured		0 (0.0%)	290 (29.6%)	
Micro-textured		0 (0.0%)	619 (63.2%)	
Macro-textured		0 (0.0%)	71 (7.2%)	
**Two-stage IBBR**				<0.001
Yes	251 (17.2%)	40 (8.4%)	211 (21.5%)	
No	1200 (82.5%)	435 (91.6%)	765 (78.1%)	
Missing	4 (0.3%)	0 (0.0%)	4 (0.4%)	
**Radiotherapy characteristics**				
**Radiotherapy before IBBR**				<0.001
No	1173 (80.6%)	383 (80.6%)	790 (80.6%)	
Two-stage IBBR (on expander)	160 (11.0%)	33 (7.0%)	127 (13.0%)	
One-stage IBBR (in case of recurrent BC)	122 (8.4%)	59 (12.4%)	63 (6.4%)	
**Radiotherapy after definitive IBBR**				0.8
Yes	1179 (81.0%)	387 (81.5%)	792 (80.8%)	
No	276 (19.0%)	88 (18.5%)	188 (19.2%)	
**Gray, median (IQR)**	50 (40-50)	50 (45.5-50)	50 (40-50.4)	1.0
**Boost**				<0.001
Yes	143 (9.8%)	42 (8.8%)	101 (10.3%)	
No	1152 (79.2%)	416 (87.6%)	736 (75.1%)	
Missing	160 (11.0%)	17 (3.6%)	143 (14.6%)	
**Radiotherapy bolus (surface)**				<0.001
Yes	165 (11.3%)	22 (4.6%)	143 (14.6%)	
No	1210 (83.2%)	432 (90.9%)	778 (79.4%)	
Missing	80 (5.5%)	21 (4.4%)	59 (6.0%)	

Abbreviations: BMI, body mass index, SSM, skin sparing mastectomy, NSM, nipple sparing mastectomy, IBBR, implant-based breast reconstruction, IQR, interquartile range, PUc, polyurethane coated; ADM, acellular dermal matrix.

a p-value between in situ and invasive.

b p-value excluding other and Missing.

The type of mastectomy differed between the groups: patients receiving polyurethane coated IBBR more frequently underwent NSM (72.2% vs. 38.7%, p < 0.001) and skin-reducing NSM (9.1% vs. 3.0%, p < 0.001), and less frequently underwent SSM (18.7% vs. 57.9%, p < 0.001). Two-stage IBBR was performed significantly less often in patients receiving polyurethane coated implants (8.4% vs. 21.5%, p < 0.001), as was the use of synthetic mesh or ADM (1.6% vs. 42.8%, p < 0.001). In the non-polyurethane coated group, 63.2% of implants were micro-textured and 29.6% were smooth textured. Among patients receiving polyurethane coated implants, the anatomical form was used in 473 cases (99.6%), whereas round-shaped implants were used in 325 cases (33.2%) in the non-polyurethane coated group (p < 0.001) (Supplementary Table 3).

RT was applied prior to the implantation of the final implant in 282 patients (overall 19.4%). Of these, 122 (overall 8.4%) had previously received RT following breast conserving surgery, while 160 (overall 11%) received RT on the expander, in case of two-stage IBBR. The majority of patients (overall 1179, 81.0%) received PMRT to the final implant. The median RT dose was 50 (IQR 40-50) Gray. Fewer patients with a polyurethane coated implant received a RT boost to the tumor bed (8.8% vs. 10.3%, p < 0.001).

The median follow-up for the entire cohort was 2.5 (IQR 1.3-3.5) years. Follow-up was longer in patients who received a polyurethane coated implant (median 2.8 years, IQR 1.5-3.6) compared to those with non-polyurethane coated implants (median 2.2 years, IQR 1.2-3.5, p < 0.001).

Use of polyurethane coated implants was associated with a lower rate of surgical revision due to capsular contracture, compared to non-polyurethane coated implants (9.3% vs. 25.7%; adjusted Hazard Ratio [aHR] 0.2, 95%CI 0.1-0.5, p < 0.001) (Fig. 2). In stratified analyses, these findings were consistent in both one-stage and two-stage IBBR groups (Table 2, Supplementary Fig. 1c and d .). In a sensitivity analysis restricted to patients without ADM or mesh, the results proved robust (Table 3, Supplementary Fig. 1a and b .).

**Fig. 2:**
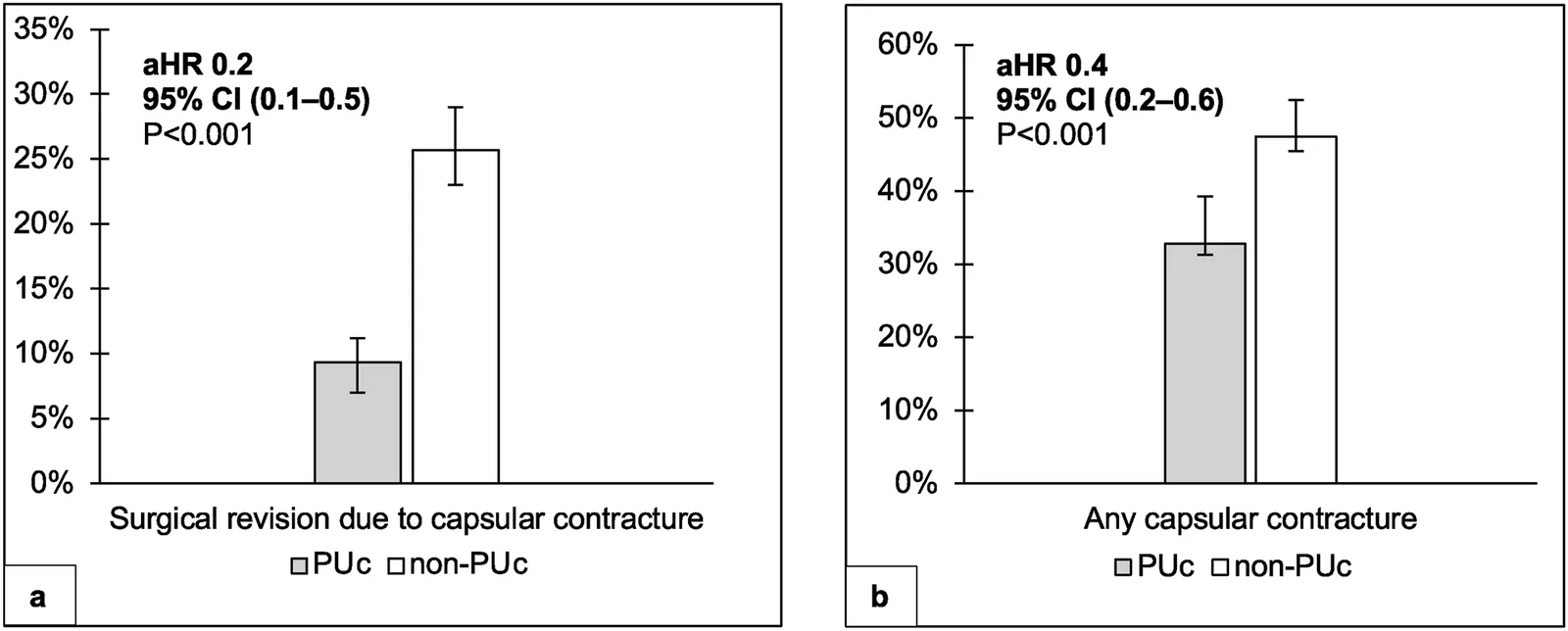
Association of type of implant (polyurethane versus non-polyurethane) with capsular contracture The whiskers represent the corresponsive 95% confidence intervals. A Rate of surgical revision due to capsular contracture in PUc and non-PUc implants. b Rate of any capsular contracture in PUc and non-PUc implants. Abbreviations: aHR, adjusted Hazard Ratio; CI, confidence interval; PUc, polyurethane coated; P, P value aHR: adjusted for age, smoking status, diabetes mellitus status, radiotherapy period, radiotherapy dose and receipt of chemotherapy.

**Table 2:** Association of type of implant (polyurethane vs. non-polyurethane) with surgical revision due to capsular contracture, stratified by one- or two-stage implant-based breast reconstruction.

Incidence of surgical revision due to CC, % (n)	PUc implants	Non-PUc implants	Adjusted HR^a^ (95% CI)	p-value
One-stage IBBR			0.3 (0.2 - 0.6)	<0.001
Yes	43 (9.9%)	213 (27.8%)		
No	392 (90.1%)	552 (72.2%)		
Two-stage IBBR			0.05 (0.003 - 0.7)	0.02
Yes	1 (2.5%)	37 (17.5%)		
No	39 (97.5%)	174 (82.6%)		

Abbreviations: CC, capsular contracture, HR, hazard ratio, CI, confidence interval, PUc, polyurethane coated, IBBR, implant-based breast reconstruction.

a For smoking, age, chemotherapy, diabetes, radiotherapy dose, radiotherapy timing (before or after IBBR).

**Table 3:** Association of type of implant (polyurethane vs. non-polyurethane) with surgical revision due to capsular contracture, stratified by use of mesh or ADM.

Incidence of surgical revision due to CC, % (n)	PUc implants	Non-PUc implants	Adjusted HR^a^ (95% CI)	p-value
With mesh or ADM			0.6 (0.07 - 4.3)	0.6
Yes	1 (12.5%)	123 (29.4%)		
No	7 (87.5%)	296 (70.6%)		
Without mesh or ADM			0.2 (0.09 - 0.6)	0.002
Yes	43 (9.2%)	127 (22.8%)		
No	423 (90.8%)	431 (77.2%)		

Abbreviations: CC, capsular contracture, ADM, acellular dermal matrix, HR, hazard ratio, CI, confidence interval, PUc, polyurethane coated.

a For smoking, age, chemotherapy, diabetes, radiotherapy dose, radiotherapy timing (before or after IBBR).

The median time from IBBR to surgical revision due to capsular contracture was 1.3 (IQR 0.7-2.2) years after IBBR with polyurethane coated implants, and 1.7 (IQR 1.1-2.6) years after IBBR with non-polyurethane coated implants. In the polyurethane group, the median time from RT to surgical revision due to capsular contracture amounted to 1.0 (IQR 0.3-1.7) year, compared to 1.4 years in the non-polyurethane group (IQR 0.8-2.2).

At two years of follow-up, Kaplan-Meier estimates indicated that 92.5% of patients with polyurethane coated implants (95%CI, 89.9-95.1%) and 81.4% of patients with non-polyurethane coated implants (95%CI, 78.7-84.2%) remained free from surgical revision due to capsular contracture (Fig. 3). In multivariable Cox proportional hazards regression analysis, the use of polyurethane coated implants was independently associated with a significantly lower hazard of surgical revision due to capsular contracture (hazard ratio [HR] 0.25; 95%CI 0.1-0.6, p < 0.001; Supplementary Fig. 2).

**Fig. 3:**
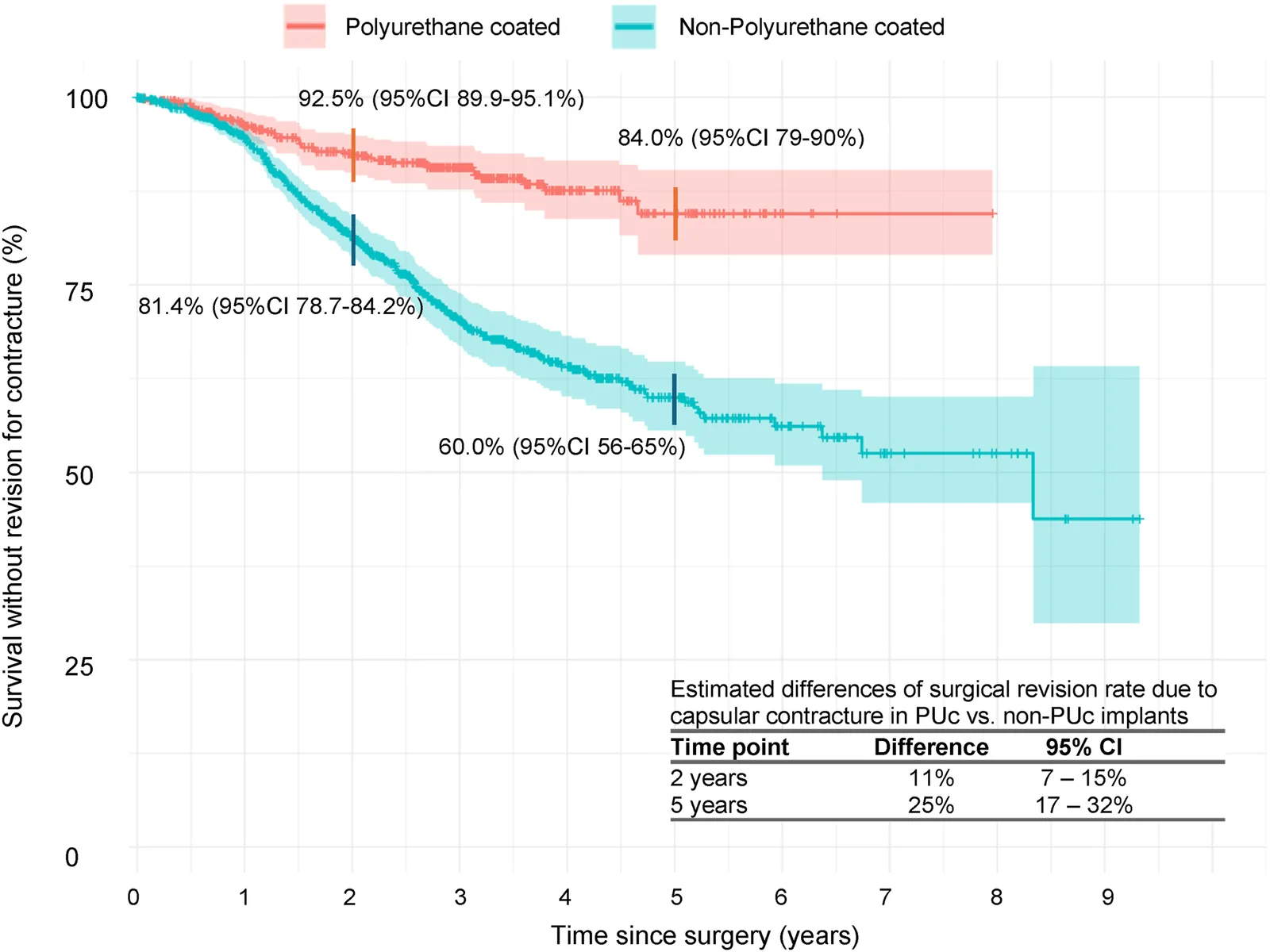
Kaplan-Meier estimates of survival without surgical revision due to capsular contracture in polyurethane coated (PUc) vs. non-PUc implants after radiotherapy, as well as estimated differences of surgical revision rate due to capsular contracture in both groups after 2 and 5 years. Abbreviations: CI, confidence interval, PUc, polyurethane coated.

In line with this finding, the rate of any capsular contracture was also significantly lower in the polyurethane group than in the non-polyurethane group (32.8% vs. 47.5%; aHR 0.4; 95%CI 0.2-0.6, p < 0.001).

Patients receiving polyurethane coated implants were less likely to experience implant loss (6.1% vs. 29.7%; aOR 0.2; 95%CI 0.09-0.4, p < 0.001), major infection (3.6% vs. 10.6%; aOR 0.3; 95%CI 0.2-0.6, p < 0.001), implant rotation or malpositioning (0.2% vs. 5.7%; aOR 0.05; 95%CI 0.0-0.7, p = 0.02), or redo surgery for any cause (22.1% vs. 41.7%; aOR 0.3; 95%CI 0.2-0.6, p = 0.001, Table 4 ).

**Table 4:** Association of type of implant (polyurethane vs. non-polyurethane) with postoperative complications.

	PUc implants n = 475	Non-PUc implants n = 980	Adjusted OR^a^ (95% CI)	p-value
**Implant loss**	29 (6.1%)	291 (29.7%)	0.2 (0.09-0.4)	<0.001
*Missing*	*17 (3.6%)*	*26 (2.6%)*		
**Hematoma**	13 (2.7%)	50 (5.1%)	0.5 (0.3-1.0)	0.06
*Missing*	*0 (0.0%)*	*3 (0.3%)*		
**Minor Infection**	29 (6.1%)	88 (9.0%)	0.8 (0.3-2.2)	0.7
*Missing*	*0 (0.0%)*	*3 (0.3%)*		
**Major Infection**	17 (3.6%)	104 (10.6%)	0.3 (0.2-0.6)	<0.001
*Missing*	*21 (4.4%)*	*10 (1.0%)*		
**Skin necrosis**	34 (7.2%)	61 (6.2%)	1.2 (0.5-2.6)	0.7
*Missing*	*0 (0.0%)*	*2 (0.2%)*		
**Wound healing disorder**	74 (15.6%)	119 (12.1%)	0.9 (0.5-1.9)	0.8
*Missing*	*0 (0.0%)*	*2 (0.2%)*		
**Seroma**	113 (23.8%)	165 (16.8%)	0.8 (0.4-1.9)	0.7
*Missing*	*39 (8.2%)*	*37 (3.8%)*		
**Implant exposure**	9 (1.9%)	63 (6.4%)	0.3 (0.1-1.2)	0.1
*Missing*	*40 (8.4%)*	*35 (3.6%)*		
**Implant rotation or malpositioning**	1 (0.2%)	56 (5.7%)	0.05 (0.004-0.7)	0.02
*Missing*	*40 (8.4%)*	*34 (3.5%)*		
**Rippling**	78 (16.4%)	99 (10.1%)	1.6 (0.8-3.4)	0.2
*Missing*	*39 (8.2%)*	*34 (3.5%)*		
**Redo surgery**	105 (22.1%)	409 (41.7%)	0.3 (0.2-0.6)	0.001
*Missing*	*0 (0.0%)*	*1 (0.1%)*		

Abbreviations: OR, odds ratio, CI, confidence interval, PUc, polyurethane coated.

a Adjusted for age, smoking status, diabetes mellitus status, radiotherapy period, radiotherapy dose and receipt of chemotherapy.

In a sensitivity analysis using the composite endpoint of surgical revision for capsular contracture or implant loss for any reason, polyurethane coated implants remained associated with a significant lower risk of the composite endpoint (aHR 0.23; 95%CI 0.13-0.40, p < 0.001), consistent with the primary analysis (Supplementary Table 4).

In addition, propensity score-based sensitivity analyses yielded results consistent with the primary mixed-effects Cox regression model (Supplementary Figs. 3 and 4, and Supplementary Tables 5–7).

## 4 Discussion

RT after IBBR is well known to be associated with substantial complications, including higher overall complication rates, increased implant loss, and elevated capsular contracture rates [25], [28], [29], [30], [31] compared with patients who do not undergo RT. The prospective multicenter iBRA study showed that PMRT significantly and relevantly worsened satisfaction and QoL in this setting [32]. Although autologous reconstruction has been associated with fewer complications and higher patient satisfaction than IBBR in the setting of PMRT [33], [34], not all patients are eligible for or wish to undergo autologous reconstruction, and IBBR therefore remains an important reconstructive option. Hence, there is a clear need to improve outcomes after IBBR in the setting of PMRT. In fact, the optimal type and timing of breast reconstruction in the setting of PMRT were identified as the two most important knowledge gaps in this field [35]. Retrospective studies have suggested that prepectoral implant placement may reduce morbidity, particularly the risk of capsular contracture, compared with subpectoral placement [25].

While the risk of capsular contracture is increased by PMRT in all patients undergoing IBBR [26], polyurethane coated implants may reduce this risk [36], [37], [38], [39].

In the present multicenter real-world study, we evaluated the risk of reoperation due to capsular contracture in patients undergoing prepectoral IBBR with either polyurethane or non-polyurethane coated implants in the setting of RT in a large retrospective cohort. Our findings demonstrated that the use of polyurethane coated implants significantly reduced the need for secondary surgery due to capsular contracture, and the risk of any capsular contracture or implant loss independently of the use of mesh/ADM or two-vs. one-stage techniques.

In non-irradiated women undergoing IBBR with polyurethane coated implants, previously published studies have reported exceptionally low rates of capsular contracture, ranging from 0.98% at 10 years to 2.19% at 25 years [40] and 2.40% at 30 years [38], [39]. These rates are substantially lower than those reported for smooth or textured implants [40], [41]. A plausible biological explanation for the reduced rates of capsular contracture observed with polyurethane coated implants relates to unique surface-tissue interactions resulting from the three-dimensional reticulated polyurethane foam architecture, which differ substantially from those observed with smooth, micro-textured, and macro-textured implant surfaces. Evidence from mechanistic studies, animal models, and clinical histopathological analyses suggests that this architecture favors early and stable tissue incorporation, limits the development of an organized circumferential capsule, and disrupts the parallel alignment of contractile elements [42], [43].

Experimental data further indicate that the protective effect of polyurethane against capsular contracture is not primarily attributable to inhibition of the fibroblast–myofibroblast transition, but instead to modulation of extracellular matrix composition, producing capsules that are thicker yet mechanically less rigid, with irregular collagen organization, lower relative type I collagen content, and a higher proportion of non-collagenous matrix despite sustained myofibroblast presence [44].

However, these same surface properties have also raised concerns regarding breast implant-associated anaplastic large cell lymphoma (BIA-ALCL), a rare complication predominantly associated with textured and polyurethane coated breast implants.

Importantly, BIA-ALCL is not exclusive to polyurethane coated implants, as cases have also been reported in association with textured implants and, more recently, in patients with an exclusively smooth implant history [45]. This broader context is relevant to the present study, in which the non-polyurethane comparator group included both textured and smooth implants. Reported cases and risk estimates also differ between polyurethane implant manufacturers. Australian registry data documented BIA-ALCL predominantly in association with Silimed polyurethane implants, with substantially fewer reported exposures involving POLYTECH polyurethane implants [46]. Notably, despite the extensive clinical use of POLYTECH polyurethane implants, only a small number of BIA-ALCL cases have been reported in association with these devices [47].

The use of mesh or ADM in prepectoral reconstruction has been hypothesized to influence capsular contracture rates after IBBR, particularly in the setting of PMRT. In the present cohort, mesh or ADM were used more frequently in the non-polyurethane group; however, multivariable analysis did not identify mesh or ADM use as being independently associated with capsular contracture. Our findings particularly suggest that the risk of surgical revision due to capsular contracture is reduced by use of polyurethane coated implants with or without the use of synthetic mesh or ADM (Supplementary Fig. 1). Consistent with these findings, a recent study including 97 patients (135 breasts) undergoing prepectoral reconstruction reported that polyurethane coated implants were a safe option for immediate breast reconstruction, with improved aesthetic outcomes and lower rates of seroma, infection, and capsular contracture compared with ADM-covered non-polyurethane coated implants [48]. Furthermore, in a retrospective analysis, polyurethane coating tended to be associated with fewer short-term complications, such as seroma and infection [21].

In the present study, two-stage IBBR was predominantly performed in the non-polyurethane group. Our findings suggest that the rate of surgical revision due to capsular contracture was lower after two-stage IBBR. Of those who underwent two-stage IBBR, 11% underwent radiotherapy while the tissue expander was in place. This is in line with other studies demonstrating lower capsular contracture rates when the tissue expander is irradiated before the definitive implant placement [49]. Although delayed reconstruction after completion of RT is sometimes considered to mitigate complications, the elevated risk of implant loss as worst-case scenario often persists [50]. Consequently, IBBR prior to PMRT remains a commonly performed practice and our data strongly suggest that this approach is associated with low capsular contracture and reoperation rates, particularly when polyurethane coated implants are used.

In the present cohort of patients with BC after RT, those who underwent IBBR with polyurethane coated implants experienced markedly lower rates of major infections, defined as those requiring surgical intervention or intravenous antibiotic therapy, as well as a reduced incidence of implant rotation or malposition, compared with patients receiving non-polyurethane coated implants. Despite the significantly lower rate of redo surgery observed in the polyurethane group (22.1% vs. 41.7%), the absolute frequency of additional surgical interventions remains clinically relevant and highlights the substantial burden of implant-based reconstruction in patients undergoing RT. Beyond implant- and technique-related factors, patient-related characteristics have also been identified as additional contributors to complication risk in patients undergoing IBBR with polyurethane coated implants. In this context, Lisa et al. reported that overweight, diabetes and autoimmune hypothyroidism were associated with minor or major complications. RT, although not associated with overall complication rates in their cohort, was significantly associated with the development of capsular contracture [51].

The primary limitation of this study is its retrospective design, which is inherently associated with the risk of selection bias and confounding. Substantial baseline differences between the polyurethane and non-polyurethane coated implant groups suggest potential selection bias, as implant selection may have been influenced by patient characteristics, surgical factors, and surgeon preferences. Furthermore, participating centers followed their own reconstructive practices and institutional standards, resulting in heterogeneity in surgical approaches and the use of mesh or acellular dermal matrices. Although center was included as a random effect and baseline differences were addressed through multivariable adjustment and an additional propensity score-based sensitivity analysis, residual confounding due to unmeasured factors, including surgeon experience and center-specific expertise, cannot be excluded. As expected in a retrospective multicenter study, data completeness and outcome ascertainment represent additional limitations. Up to 8% of data were missing on some secondary endpoints, and minor events may have been underreported. Moreover, subsequent surgical procedures were retrospectively reported by investigators at the participating centers, complete ascertainment of revision procedures performed at other institutions could not be ensured. This may have resulted in an underestimation of revision rates, particularly for procedures occurring later during follow-up, and differential underreporting between implant groups cannot be excluded.

The relatively short median follow-up period differed between the two groups and further limits the interpretation of long-term outcomes. However, follow-up was longer in the polyurethane group, making it unlikely that the lower observed rates of surgical revision due to capsular contracture can be explained by insufficient observation time. Long-term outcomes were not assessed in this study, and the available follow-up was insufficient to evaluate rare, late-onset complications such as BIA-ALCL.

Furthermore, RT could not be evaluated in detail because the information on number of fractions was not available. Although the median total dose of 50 Gy in both groups suggests predominantly conventional fractionation, differences in fractionation between groups, reflecting the increasing use of hypofractionated regimens during the study period, cannot be excluded.

Finally, patient-reported outcomes and aesthetic assessments were not systematically collected, limiting conclusions regarding quality of life and cosmetic outcomes. Despite these limitations, this study has several strengths. Firstly, it represents the largest study to date comparing outcomes between prepectoral polyurethane and non-polyurethane coated implants in patients receiving RT. The consistency of the observed findings across multivariable models, subgroup analyses, and sensitivity analyses lends support to the main findings, although residual confounding remains possible. Finally, the large number of participating sites across different healthcare settings and geographic regions increases the generalizability and applicability of the findings.

Nevertheless, as a retrospective analysis, our findings warrant confirmation in the prospective setting. Ongoing prospective studies, such as the international I-PREPARE registry [52], may provide valuable prospective data on immediate prepectoral IBBR in the setting of RT. Further dedicated prospective studies with longer follow-up are needed to assess the long-term effectiveness and safety in both groups, including, for example, the occurrence of ALCL.

## 5 Conclusions

The present study showed a strong association between use of polyurethane vs. non-polyurethane coated implants and lower likelihood of surgical revision due to capsular contracture, as well as lower risk of complications, in the setting of RT. These findings may inform implant selection and reconstructive planning for patients requiring RT.

## CRediT authorship contribution statement

**Florian Fitzal:** Writing – review & editing, Supervision, Methodology, Investigation, Conceptualization. **Rama Kiblawi:** Writing – original draft, Investigation, Formal analysis, Data curation, Conceptualization. **Kerstin Wimmer:** Writing – review & editing, Writing – original draft, Visualization, Resources. **Florian S. Halbeisen:** Methodology, Formal analysis. **Gianluca Franceschini:** Writing – review & editing, Data curation. **Lorenzo Scardina:** Writing – review & editing, Data curation. **Fabio Marazzi:** Writing – review & editing, Data curation. **Simona Gipe:** Writing – review & editing, Data curation. **Cynthia Kohl:** Writing – review & editing, Data curation. **Franziska Trapp:** Writing – review & editing, Data curation. **Maja Molska:** Writing – review & editing, Data curation. **Oliwia Bednarek:** Data curation. **Martin Heidinger:** Writing – original draft, Supervision, Methodology. **Edvin Ostapenko:** Writing – review & editing. **Amit Agrawal:** Writing – review & editing, Data curation. **Konstantina Balafa:** Writing – review & editing, Data curation. **Maggie Banys-Paluchowski:** Writing – review & editing, Data curation. **Justin M. Broyles:** Writing – review & editing, Data curation. **Oreste D. Gentilini:** Writing – review & editing, Data curation. **Maite Goldschmidt:** Project administration. **Eduardo González:** Writing – review & editing, Data curation. **Martin Haug:** Resources, Data curation. **Elisabeth A. Kappos:** Writing – review & editing, Data curation. **Michael Knauer:** Writing – review & editing, Visualization, Data curation. **Giacomo Montagna:** Writing – review & editing, Visualization, Data curation, Writing – original draft, Data curation. **Ira Oikonomou:** Investigation, Data curation. **Martín Rivas Ibarra:** Writing – review & editing, Data curation. **Simone Ross-Keck:** Project administration. **Dirk J. Schaefer:** Writing – review & editing, Resources, Data curation, Conceptualization. **Andy Schneider:** Project administration. **Nicole Speck:** Writing – review & editing, Data curation. **Dario Virzi:** Writing – review & editing, Data curation. **Mateusz Wichtowski:** Writing – review & editing, Data curation. **Artefaa Al-Shamari:** Writing – review & editing, Data curation. **Mowid Hamad:** Writing – review & editing, Data curation. **André Pfob:** Writing – review & editing, Data curation. **Jörg Heil:** Writing – review & editing, Data curation. **Lisa Stenman Skarsgård:** Data curation. **Christian Korvald:** Writing – review & editing, Visualization, Data curation. **Daniele Passeri:** Data curation. **Mariacristina Toffanin:** Writing – review & editing, Data curation. **Massimo Ferrucci:** Writing – review & editing, Visualization, Supervision, Methodology, Data curation. **Walter P. Weber:** Writing – review & editing, Writing – original draft, Visualization, Validation, Supervision, Methodology, Investigation, Conceptualization. **Khaled Abdelwahab:** Resources. **Guldeniz Karadeniz Cakmak:** Resources. **Francesco Caruso:** Resources. **Sabatino D'Archi:** Resources. **Rosa Di Micco:** Resources. **Annasilvia Di Pumpo:** Resources. **Salahddin A. Gehani:** Resources. **Camila Gutierrez Neira:** Resources. **Laura Henderson:** Resources. **Tari A. King:** Resources, Writing – review & editing. **Jaime Letzkus Berrios:** Resources. **Maciej Łukaszek:** Resources. **Zoltan Matrai:** Resources. **Petra Palkó:** Resources. **Eleonora Petrazzuolo:** Resources. **Valentina Pezzetti:** Resources. **Pier Carlo Rassu:** Resources. **Anne Marie Raymakers:** Resources. **Nicola Rocco:** Resources. **Nicole Rotmensz:** Resources. **Tina Schnitzbauer:** Resources. **Nikolas Tauber:** Resources. **José H. Volders:** Resources, Writing – review & editing. **Denise Vorburger:** Resources.

## Meeting presentation

This paper was presented orally at the annual meeting of the Society of Surgical Oncology in Phoenix, Arizona on March 5th – 7th, 2026. Moreover, this work was presented during the Opening Ceremony at the 15th European Breast Cancer Conference in Barcelona, Spain on the 25th of March 2026.

## Data sharing statement

The datasets used and/or analyzed during the current study are available from the corresponding author upon reasonable request.

## Declarationof generative AI and AI-assisted technologies in the manuscript preparation process

During the preparation of this work, the authors used Claude (Anthropic) to edit the text for language and grammar. The authors reviewed and edited the output as needed and take full responsibility for the content of the published article.

## Funding/support

No external funding has been obtained for this study.

## Declaration of competing interest

The authors declare the following financial interests/personal relationships which may be considered as potential competing interests: Walter P. Weber reports a relationship with Oncoplastic Breast Consortium GmbH that includes: board membership as Founder/Chairman. Walter P. Weber reports a relationship with Agendia Inc that includes: funding grants. Walter P. Weber reports a relationship with MSD that includes: speaking and lecture fees. Florian Fitzal reports a relationship with AstraZeneca Pharmaceuticals LP that includes: funding grants and paid expert testimony. Florian Fitzal reports a relationship with F. Hoffmann-La Roche Ltd that includes: funding grants and paid expert testimony. Florian Fitzal reports a relationship with Novartis Pharmaceuticals Corporation that includes: funding grants and paid expert testimony. Florian Fitzal reports a relationship with Stemline Therapeutics Inc that includes: paid expert testimony. Florian Fitzal reports a relationship with Bondimed that includes: funding grants. Amit Agrawal reports a relationship with NIHR Cambridge Biomedical Research Centre that includes: funding grants. Amit Agrawal reports a relationship with Addenbrooke's Charitable Trust grant that includes: funding grants. Amit Agrawal reports a relationship with National Institute for Health and Care Research that includes: funding grants. Amit Agrawal reports a relationship with Cancer Research UK Primer award that includes: funding grants. Oreste Gentilini reports a relationship with Applied Medical Resources Corp that includes: speaking and lecture fees. Oreste Gentilini reports a relationship with MSD that includes: speaking and lecture fees. Oreste Gentilini reports a relationship with AstraZeneca Pharmaceuticals LP that includes: speaking and lecture fees. Oreste Gentilini reports a relationship with Eli Lilly and Company that includes: speaking and lecture fees. Oreste Gentilini reports a relationship with Bayer AG that includes: speaking and lecture fees. Simona Gipe reports a relationship with Novartis Pharmaceuticals Corporation that includes: speaking and lecture fees and travel reimbursement. Simona Gipe reports a relationship with AstraZeneca that includes: consulting or advisory and speaking and lecture fees. Simona Gipe reports a relationship with MSD that includes: speaking and lecture fees and travel reimbursement. Simona Gipe reports a relationship with F. Hoffmann-La Roche Ltd that includes: consulting or advisory and speaking and lecture fees. Simona Gipe reports a relationship with Gilead Sciences Inc that includes: speaking and lecture fees. Simona Gipe reports a relationship with Daiichi that includes: travel reimbursement. Tina Schnitzbauer reports a relationship with F. Hoffmann-La Roche Ltd that includes: speaking and lecture fees. Tina Schnitzbauer reports a relationship with Pfizer Inc that includes: speaking and lecture fees. Tina Schnitzbauer reports a relationship with AstraZeneca Pharmaceuticals LP that includes: speaking and lecture fees. Tina Schnitzbauer reports a relationship with MSD that includes: travel reimbursement. Franziska Trapp reports a relationship with Menarini that includes: speaking and lecture fees and travel reimbursement. Cynthia Kohl reports a relationship with AstraZeneca Pharmaceuticals LP that includes: speaking and lecture fees. If there are other authors, they declare that they have no known competing financial interests or personal relationships that could have appeared to influence the work reported in this paper. Given his role as Editor, Jörg Heil had no involvement in the peer-review of this article and has no involvement in the peer review of this article and has no access to information regarding its peer review.
