# Supplementary material for: Use of prepectoral polyurethane coated implants in the setting of radiotherapy: the OPBC-09 PRExRT retrospective cohort study
Source: Breast. 2026 Oct 30;90:104951. doi: 10.1016/j.breast.2026.104951 (PMC13769591; doi:10.1016/j.breast.2026.104951)
Supplement: Supplementary file 1 [file mmc1.docx]

**Supplementary material**

Supplementary Table 1: Participating sites

| **Site** | **Department** |
| --- | --- |
| Argentina, Buenos Aires | Department of Mastology, Ángel H. Roffo Institute of Oncology, University of Buenos Aires, Buenos Aires, Argentina |
| Austria, Vienna | Department of Surgery and Comprehensive Cancer Centre, Medical University of Vienna, Vienna, Austria |
| Chile, Santiago de Chile | Department of Breast Surgery, Arturo López Pérez Foundation, Santiago, Chile |
| Chile, Santiago de Chile | Department of Breast Surgery, San Borja Arriaran Clinical Hospital, University of Chile, Chile |
| Egypt, Mansoura | Mansoura Oncology Centre, Surgical Oncology Department, Faculty of Medicine, Mansoura University, Mansoura, Egypt |
| Germany, Essen | Interdisciplinary Breast Centre, Kliniken Essen-Mitte, Essen, Germany |
| Germany, Heidelberg | Department of Gynecology and Obstetrics, Breast Unit, Heidelberg University Hospital, Heidelberg, Germany |
| Germany, Luebeck | Department of Gynecology and Obstetrics, University Hospital Schleswig-Holstein, Campus Lübeck, Lübeck, Germany |
| Italy, Catania | Breast Surgery Unit, Humanitas Istituto Clinico Catanese, Misterbianco, Catania, Italy |
| Italy, Milano | Breast Surgery Unit, San Raffaele Scientific and Research Hospital, Milan, Italy |
| Italy, Novi Ligure | General Surgery Department, San Giacomo Hospital, Novi Ligure, Alessandria, Italy |
| Italy, Naples | Breast Department, G. Pascale National Cancer Institute, and Department of Advanced Biomedical Sciences, University Federico II, Naples, Italy |
| Italy, Padova | Breast Surgical Oncology Unit, Istituto Oncologico Veneto IRCCS, Padua, Italy |
| Italy, Roma | Breast Unit, Department of Science and Health of Women, Children and Public Health, Fondazione Policlinico Universitario Agostino Gemelli IRCCS, Rome, Italy |
| Netherlands, Utrecht | Diakonessenhuis Utrecht, Department of Surgical Oncology, Utrecht, Netherlands |
| Norway, Oslo | Oslo University Hospital, Department of Plastic and Reconstructive Surgery, Oslo, Norway |
| Poland, Poznan | Department of Surgical Oncology, Institute of Oncology, Poznan University of Medical Sciences Szamarzewskiego 84, 60-569 Poznan, Poland |
| Poland, Zielona Góra | University Hospital in Zielona Góra, Clinical Department of General and Oncological Surgery, Zielona Góra, Poland |
| Qatar, Doha | Hamad Medical Corporation, Department of Oncoplastic Breast Surgery, Doha, Qatar |
| Sweden, Stockholm | Södersjukhuset AB, Division of Breast Surgery, Department of General Surgery, Stockholm, Sweden |
| Switzerland, Basel | University of Basel and Breast Clinic, University Hospital Basel, Basel, Switzerland |
| Switzerland, St. Gallen | Tumor- und BrustZentrum Ostschweiz, Breast Centre, St. Gallen, Switzerland |
| Switzerland, Zurich | Breast Cancer Centre, Department of Gynecology, University Hospital Zurich, Zurich, Switzerland |
| Turkey, Zonguldak | Department of Surgery, Zonguldak Bulent Ecevit University, The School of Medicine, Zonguldak, Turkey |
| UK, Cambridge | Department of Breast Surgery, Cambridge University Hospitals, Cambridge, UK |
| USA, Boston | Division of Breast Surgery, Brigham and Women's Hospital, Dana Farber/Brigham Cancer Centre, Boston, MA, USA |

Supplementary Table 2a: Breast implant manufacturers by participating site

| **Site** | **Breast Implant manufacturer** |
| --- | --- |
| Argentina, Buenos Aires | Microthane®; Polytech Health and Aesthetics GmbH |
| Austria, Vienna | Microthane®; Polytech Health and Aesthetics GmbH |
| Chile, Santiago de Chile, Arturo López Pérez Foundation | MENTOR™; Mentor Worldwide LLC |
| Chile, Santiago de Chile, San Borja Arriaran Clinical Hospital | Nagor®, Nagor Ltd., GC Aesthetics |
| Egypt, Mansoura | Microthane®; Polytech Health and Aesthetics GmbH |
| Germany, Essen | Microthane®; Polytech Health and Aesthetics GmbH |
| Germany, Heidelberg | Microthane®; Polytech Health and Aesthetics GmbH |
| Germany, Luebeck | Sebbin Integrity®; Sebbin SAS and  MENTOR™; Mentor Worldwide LLC |
| Italy, Catania | Microthane®; Polytech Health and Aesthetics GmbH |
| Italy, Milano | Microthane®; Polytech Health and Aesthetics GmbH |
| Italy, Novi Ligure | Microthane®; Polytech Health and Aesthetics GmbH |
| Italy, Naples | MENTOR™; Mentor Worldwide LLC |
| Italy, Padova | MENTOR™; Mentor Worldwide LLC and  Microthane®; Polytech Health and Aesthetics GmbH |
| Italy, Roma | Microthane®; Polytech Health and Aesthetics GmbH |
| Netherlands, Utrecht | MENTOR™; Mentor Worldwide LLC |
| Norway, Oslo | MENTOR™; Mentor Worldwide LLC and  Sebbin Integrity®; Sebbin SAS |
| Poland, Poznan | Microthane®; Polytech Health and Aesthetics GmbH |
| Poland, Zielona Góra | Microthane®; Polytech Health and Aesthetics GmbH |
| Qatar, Doha | MENTOR™; Mentor Worldwide LLC |
| Sweden, Stockholm | MENTOR™; Mentor Worldwide LLC |
| Switzerland, Basel | MENTOR™; Mentor Worldwide LLC and  Motiva Implants®; Establishment Labs® S.A. |
| Switzerland, St. Gallen | GCA® breast implants, GC Aesthetics, Ltd. and  Motiva Implants®; Establishment Labs® S.A. |
| Switzerland, Zurich | Motiva Implants®; Establishment Labs® S.A. and  Microthane®; Polytech Health and Aesthetics GmbH |
| Turkey, Zonguldak | MENTOR™; Mentor Worldwide LLC |
| UK, Cambridge | MENTOR™; Mentor Worldwide LLC |
| USA, Boston | Natrelle®; Allergan, AbbVie |

Supplementary Table 2b: Synthetic mesh and acellular dermal matrix manufacturers by participating site

| **Site** | **Synthetic mesh and acellular dermal matrix manufacturer** |
| --- | --- |
| Argentina, Buenos Aires | Not used |
| Austria, Vienna | Not used |
| Chile, Santiago de Chile, Arturo López Pérez Foundation | TiLOOP® Bra Pocket, pfm medical ag |
| Chile, Santiago de Chile, San Borja Arriaran Clinical Hospital | VICRYL™ mesh, Ethicon, Inc., Johnson & Johnson |
| Egypt, Mansoura | Not used |
| Germany, Essen | Not used |
| Germany, Heidelberg | Not used |
| Germany, Luebeck | Not used |
| Italy, Catania | Not used |
| Italy, Milano | Not used |
| Italy, Novi Ligure | Not used |
| Italy, Naples | Not used |
| Italy, Padova | TiLOOP® Bra Pocket, pfm medical ag and  Braxon® acellular dermal matrix, DECOmed S.r.l. |
| Italy, Roma | Not used |
| Netherlands, Utrecht | TIGR® Matrix Surgical Mesh, Novus Scientific AB |
| Norway, Oslo | TiLOOP® Bra Pocket, pfm medical ag and  VICRYL™ knitted mesh, Ethicon, Inc., Johnson & Johnson |
| Poland, Poznan | Not used |
| Poland, Zielona Góra | Not used |
| Qatar, Doha | Braxon® acellular dermal matrix, DECOmed S.r.l. |
| Sweden, Stockholm | TIGR® Matrix Surgical Mesh, Novus Scientific AB |
| Switzerland, Basel | SurgiMend® PRS Meshed, Integra LifeSciences Corporation |
| Switzerland, St. Gallen | TiLOOP® Bra Pocket, pfm medical ag |
| Switzerland, Zurich | TiLOOP® Bra Pocket, pfm medical ag |
| Turkey, Zonguldak | Braxon® acellular dermal matrix, DECOmed S.r.l. |
| UK, Cambridge | Braxon® and Native® acellular dermal matrix, DECOmed S.r.l. and  SurgiMend® PRS Meshed, Integra LifeSciences Corporation |
| USA, Boston | ALLODERM™, LifeCell Corporation, AbbVie |

Supplementary Table 3: Additional patient, tumour and treatment characteristics

|  | **Overall study population**  **n=1455** | **PUc implants**  **n=475** | **Non-PUc implants**  **n=980** | **p-value** |
| --- | --- | --- | --- | --- |
| **Invasive cancer subtype^a^** |  |  |  | <0.001 |
| Lobular | 268 (18.4%) | 71 (14.9%) | 197 (20.1%) |  |
| Ductal | 1109 (76.2%) | 381(80.2%) | 728 (74.3%) |  |
| Micropapillary | 12 (0.8%) | 0 (0.0%) | 12 (1.2%) |  |
| Other | 28 (1.9%) | 4 (0.8%) | 24 (2.4%) |  |
| Missing | 38 (2.6%) | 19 (4.0%) | 19 (1.9%) |  |
| **Laterality** |  |  |  | 0.28 |
| Right | 705 (48.4%) | 232 (48.8%) | 473 (48.3%) |  |
| Left | 744 (51.1%) | 243 (51.2%) | 501 (51.1%) |  |
| Bilateral | 6 (0.4%) | 0 (0.0%) | 6 (0.6%) |  |
| **Estrogen Receptor^a^** |  |  |  | 0.06 |
| Positive | 1104 (75.9%) | 360 (75.8%) | 744 (75.9%) |  |
| Negative | 302 (20.8%) | 81 (17.0%) | 221 (22.6%) |  |
| Missing | 49 (3.4%) | 34 (7.2%) | 15 (1.5%) |  |
| **Progesteron Receptor^a^** |  |  |  | 0.02 |
| Positive | 945 (64.9%) | 315 (66.3%) | 630 (64.3%) |  |
| Negative | 461 (31.7%) | 126 (26.5%) | 335 (34.2%) |  |
| Missing | 49 (3.4%) | 34 (7.2%) | 15 (1.5%) |  |
| **HER2 status^a^** |  |  |  | <0.001 |
| Positive | 308 (21.2%) | 68 (14.3%) | 240 (24.5%) |  |
| Negative | 1083 (74.4%) | 368 (77.5%) | 715 (73.0%) |  |
| Missing | 64 (4.4%) | 39 (8.2%) | 25 (2.6%) |  |
| **Grade^a^** |  |  |  | 0.03 |
| 1 | 77 (5.3%) | 33 (6.9%) | 44 (4.5%) |  |
| 2 | 715 (49.1%) | 209 (44.0%) | 506 (51.6%) |  |
| 3 | 554 (38.1%) | 154 (32.4%) | 400 (40.8%) |  |
| Missing | 109 (7.5%) | 79 (16.6%) | 30 (3.1%) |  |
| **Implant form** |  |  |  | <0.001 |
| Round | 327 (22.5%) | 2 (0.42%) | 325 (33.16%) |  |
| Anatomic | 1120 (77.0%) | 473 (99.6%) | 647 (66.02%) |  |
| Missing | 8 (0.6%) | 0 (0.0%) | 8 (0.82%) |  |
| **Implant volume,** ml, median (IQR) | 350 (265 to 425) | 342.5 (265 to 420) | 350 (255 to 430) | 0.80 |
| **Drainage** |  |  |  | <0.001 |
| Yes | 1348 (92.6%) | 464 (97.7%) | 884 (90.2%) |  |
| No | 100 (6.9%) | 8 (1.7%) | 92 (9.4%) |  |
| Missing | 7 (0.5%) | 3 (0.6%) | 4 (0.4%) |  |
| **Systemic treatment characteristics** | | | | |
| **Neoadjuvant Chemotherapy** |  |  |  | 0.04 |
| Yes | 789 (54.2%) | 239 (50.3%) | 550 (56.1%) |  |
| No | 666 (45.8%) | 236 (49.7%) | 430 (43.9%) |  |
| **Adjuvant Chemotherapy** |  |  |  | 0.23 |
| Yes | 576 (39.6%) | 199 (41.9%) | 377 (38.5%) |  |
| No | 879 (60.4%) | 276 (58.1%) | 603 (61.5%) |  |
| **Adjuvant Endocrine Therapy** |  |  |  | 0.13 |
| Yes | 1068 (73.4%) | 364 (76.6%) | 704 (71.8%) |  |
| No | 381 (26.2%) | 109 (23.0%) | 272 (27.8%) |  |
| Missing | 6 (0.4%) | 2 (0.4%) | 4 (0.4%) |  |
| **Other systemic therapies** |  |  |  | <0.001 |
| Yes | 507 (34.8%) | 166 (34.9%) | 341 (34.8%) |  |
| No | 911 (62.6%) | 283 (59.6%) | 628 (64.1%) |  |
| Missing | 37 (2.5%) | 26 (5.5%) | 11 (1.1%) |  |

^a^ p-value excluding other and Missing

Abbreviations: n, number; IQR, interquartile range; PUc, polyurethane coated

Supplementary Table 4: Association of type of implant (polyurethane vs. non-polyurethane) with surgical revision due to capsular contracture or any implant loss

| **Surgical revision due to CC or implant loss, % (n)** | **PUc** | **Non-PUc** | **Adjusted HR***  **(95% CI)** | **p-value** |
| --- | --- | --- | --- | --- |
|  |  |  | 0.23 (0.13 - 0.40) | <0.001 |
| Yes | 60 (12.47%) | 441 (40.72%) |  |  |
| No | 421 (87.53%) | 642 (59.28%) |  |  |

*for smoking, age, chemotherapy, diabetes, radiotherapy dose, radiotherapy timing (before or after IBBR)

Abbreviations: CC, capsular contracture, ADM, acellular dermal matrix, HR, hazard ratio, CI, confidence interval, PUc, polyurethane coated

Supplementary Table 5: Covariate balance before and after weighting

| **Variable** | **Non-PUc (unw)** | **PUc**  **(unw)** | **SMD (unw)** | **Non-PUc (ATE)** | **PUc**  **(ATE)** | **SMD (ATE)** | **SMD**  **(ATO)** |
| --- | --- | --- | --- | --- | --- | --- | --- |
| No Mesh or ADM | 57.10 | 98.30 | 0.41 | 70.80 | 73.40 | 0.03 | <0.001 |
| Two-stage IBBR (No) | 78.40 | 91.60 | 0.13 | 83.30 | 90.40 | 0.07 | <0.001 |
| Smoking: Ongoing | 10.10 | 18.10 | 0.08 | 12.50 | 11.20 | 0.01 | <0.001 |
| Smoking: Never | 82.50 | 69.90 | 0.13 | 78.30 | 80.50 | 0.02 | <0.001 |
| Smoking: Ever | 7.50 | 12.00 | 0.04 | 9.20 | 8.40 | 0.01 | <0.001 |
| Diabetes (No) | 96.00 | 93.90 | 0.02 | 95.10 | 95.70 | 0.01 | <0.001 |
| Radiotherapy after | 80.70 | 81.50 | 0.01 | 81.60 | 86.80 | 0.05 | <0.001 |
| Adjuvant chemotherapy (No) | 61.50 | 58.10 | 0.03 | 60.50 | 56.00 | 0.05 | <0.001 |
| Neoadjuvant chemotherapy (No) | 43.90 | 49.70 | 0.06 | 45.80 | 40.40 | 0.05 | <0.001 |
| Age at surgery, y  (SD) | 47.10 (10.2) | 48.10  (9.90) | 0.10 | 47.48 (10.20) | 46.20 (9.89) | 0.13 | <0.001 |
| Radiation dose, Gy  (SD) | 45.80 (6.70) | 47.70  (4.60) | 0.33 | 46.38  (6.60) | 47.05 (4.85) | 0.12 | <0.001 |

Values are presented as percentages or mean (SD), as appropriate.

Abbreviations: ADM, acellular dermal matrix; IBBR, implant-based breast reconstruction; PUc, polyurethane coated implants; unw, unweighted; y, years; Gy, Gray; SD, standard deviation; ATE, Average Treatment Effect; ATO, Average Treatment Effect in the Overlap population; prop.score, propensity score; SMD, standardized mean difference.

Supplementary Table 6: Weight diagnostics of the main analysis

| **Method** | **ESS Non-PUc** | **ESS PUc** | **Max Weight** |
| --- | --- | --- | --- |
| IPTW (ATE) | 841.33 | 77.03 | 69.87 |
| Overlap weighting (ATO) | 532.99 | 441.87 | 0.99 |

Conventional IPTW resulted in several extreme weights and a reduced effective sample size in the PUc implant group, indicating limited overlap between groups. Overlap weighting (ATO) achieved excellent covariate balance while maintaining a substantially larger effective sample size.

Abbreviations: IPTW, Inverse Probability of Treatment Weighting; ESS, Effective Sample Size; PUc, polyurethane coated implants; ATE, Average Treatment Effect; ATO, Average Treatment Effect in the Overlap population.

Supplementary Table 7: Sensitivity analysis of the primary endpoint with inverse probability of treatment weighting

| **Method** | **HR** | **95% CI** | **p-value** |
| --- | --- | --- | --- |
| Mixed-effect Cox | 0.24 | 0.12 - 0.51 | <0.001 |
| IPTW (ATE) | 0.43 | 0.18 - 1.04 | 0.06 |
| Overlap weighting (ATO) | 0.35 | 0.17 - 0.75 | 0.01 |

The propensity score-based analyses yielded effect estimates consistent with the primary mixed-effects Cox regression model, supporting the robustness of the main findings despite baseline differences between groups.

Abbreviations: HR, hazard ratio; CI, confidence interval; IPTW, Inverse Probability of Treatment Weighting; ATE, Average Treatment Effect; ATO, Average Treatment Effect in the Overlap population.

Supplementary Figure 1: Association of type of implant (polyurethane vs. non-polyurethane) with surgical revision due to capsular contracture, stratified by use of synthetic mesh or acellular dermal matrix, and one- or two-stage implant-based breast reconstruction

a. **Stratified by use of synthetic mesh or ADM**
b. **Stratified by no use of synthetic mesh or ADM**
d. **Stratified by two-stage IBBR**
c. **Stratified by one-stage IBBR**

**aHR 0.2**

**95% CI (0.1–0.5)**

p<0.001

**aHR 0.4**

**95% CI (0.05–3.0)**

p=0.358

**aHR 0.3**

**95% CI (0.1–0.5)**

p<0.001

**aHR 0.1**

**95% CI (0.02–1.2)**

p=0.079

The whiskers represent the corrensponding 95% confidence intervals.

Hazard ratios were estimated using a multivariable Cox proportional hazards regression model. The model was adjusted for age, smoking status, diabetes mellitus status, radiotherapy period, radiotherapy dose and receipt of chemotherapy.

**a.** Among those with synthetic mesh or ADM, 1 out of 8 women (12%) with PUc versus 123 of 419 (29%) with non-PUc required a surgical revision due to CC.

**b.** Among those without synthetic mesh or ADM, 43/467 women (9%) in the PUc group versus 127/558 (23%) in the non-PUc group required a surgical revision due to CC.

**c.** Among those who underwent one-stage IBBR, 43/435 women (10%) in the PUc group versus 213/765 (28%) in the non-PUc group required a surgical revision due to CC.

**d.** Among those who underwent two-stage IBBR, 1/40 (0.2%) in the PUc group versus 37/211 (1.8%) in the non-PUc group required a surgical revision due to CC.

Abbreviations: ADM, acellular dermal matrix; aHR, adjusted Hazard Ratio; CC, capsular contracture; PUc, polyurethane coated implants; p, p-value

aHR: adjusted for age, smoking status, diabetes mellitus status, radiotherapy period, radiotherapy dose and receipt of chemotherapy.

HR	95% CI	p

0	1	2	3	4

Supplementary Figure 2: Forest plot of the multivariable Cox proportional hazards regression analysis for surgical revision due to capsular contracture

Abbreviations: HR, hazard ratio; CI, confidence interval; p, p-value; ADM, acellular dermal matrix; RT, radiotherapy; IBBR, implant-based breast reconstruction; Ref, reference

Supplementary Figure 3: Propensity score distribution by implant group

PUc

Non-PUc

Abbreviations: ATE, Average Treatment Effect; ATO, Average Treatment Effect in the Overlap population; PUc, polyurethane coated implants

 Supplementary Figure 4: Covariate balance before and after propensity score weighting

Abbreviations: ADM, acellular dermal matrix, ATE, Average Treatment Effect; ATO, Average Treatment Effect in the Overlap population
